# Challenges in the Therapeutic Targeting of KCa Channels: From Basic Physiology to Clinical Applications

**DOI:** 10.3390/ijms25052965

**Published:** 2024-03-04

**Authors:** Nhung Thi Hong Van, Woo Kyung Kim, Joo Hyun Nam

**Affiliations:** 1 Department of Physiology, Dongguk University College of Medicine, Gyeongju 38066, Republic of Korea; vthn0295@gmail.com; 2 Channelopathy Research Center (CRC), Dongguk University College of Medicine, Goyang 10326, Republic of Korea; 3 Department of Internal Medicine, Graduate School of Medicine, Dongguk University, Goyang 10326, Republic of Korea

**Keywords:** KCa channels, BK channels, IK channels, SK channels, KCa channel modulators

## Abstract

Calcium-activated potassium (KCa) channels are ubiquitously expressed throughout the body and are able to regulate membrane potential and intracellular calcium concentrations, thereby playing key roles in cellular physiology and signal transmission. Consequently, it is unsurprising that KCa channels have been implicated in various diseases, making them potential targets for pharmaceutical interventions. Over the past two decades, numerous studies have been conducted to develop KCa channel-targeting drugs, including those for disorders of the central and peripheral nervous, cardiovascular, and urinary systems and for cancer. In this review, we synthesize recent findings regarding the structure and activating mechanisms of KCa channels. We also discuss the role of KCa channel modulators in therapeutic medicine. Finally, we identify the major reasons behind the delay in bringing these modulators to the pharmaceutical market and propose new strategies to promote their application.

## 1. Introduction

The relationship between calcium ions and potassium permeability in human erythrocytes was first recognized in 1958 when Gárdos found that the presence of calcium increased potassium permeability [1]. Then, in 1972, Ca^2+^-activated K^+^ (KCa) currents were reported in various types of neurons in mollusks, vertebrates, and humans [2,3]. Like other potassium channels, KCa channels participate in the determination of resting potential in living cells. However, because of their relationship with calcium, an important second messenger, KCa channels also play an important role in controlling membrane excitability and cell volume in non-excitable cells [4]. Based on their single-channel conductance, KCa channels can be categorized into three main subfamilies: large- (BK; 200–300 pS), intermediate- (IK; 30–40 pS), and small-conductance (SK; 4–14 pS) channels [5,6,7]. The first subfamily comprises solely BK channels, also known as KCa1.1, Maxi-K, or Slo1. The intermediate-conductance subfamily includes IK channels, alternatively referred to as KCa3.1, SK4, or IK1. The final subfamily contains three members, SK1, SK2, and SK3, which correspond to KCa2.1, KCa2.2, and KCa2.3, respectively [8]. BK channels open due to increases in cytosolic free Ca^2+^ or membrane depolarization, whereas IK and SK channels are activated only by intracellular free Ca^2+^ [9,10,11]. Significant differences exist between the BK, IK, and SK channels in terms of Ca^2+^-binding affinities and sites. BK channels have a low Ca^2+^-binding affinity of about 1–11 μM; however, IK and SK channels have high affinities with Ca^2+^ of approximately 0.1–0.4 μM and 0.3–0.75 μM, respectively [12,13]. Calcium ions directly open BK channels through the regulator of conductance of K^+^ (RCK) structural domains and activate IK and SK channels through a calmodulin-binding domain [10,14,15].

Structurally, KCa channels are composed of a tetramer of α subunits [6,8,10]. The α subunits of BK channels are encoded by *KCNMA1* [16]. The α subunits of SK1, SK2, and SK3 channels are encoded by *KCNN1*, *KCNN2*, and *KCNN3*, respectively. These genes were first cloned in 1996 and show high homology in their transmembrane domains [17]. In 1997, *KCNN4*, which encodes the α subunits of IK channels, was cloned and found to exhibit a 41–42% similarity at the amino acid level with SK channels [18]. Due to structural similarities, IK channels are now classified in the same subfamily as SK channels [19]. The properties of the KCa channels are listed in Table 1.

The unique structure of KCa channels allows them to be activated by intracellular Ca^2+^ and induce membrane potential (in the case of BK channels), making them pivotal in the repolarization and hyperpolarization of cellular membranes, as well as in the regulation of cytosolic Ca^2+^, a secondary messenger. As a result, KCa channels play crucial roles in fundamental physiological activities and have become attractive targets for interventions for physiological disorders. Many KCa channel activators and inhibitors have been reported in vitro, in vivo, and in clinical trials. In this review, we synthesize the available knowledge regarding the structures, distributions, and biological functions of KCa channels and their modulators in the context of potential therapeutic drugs. We also discuss important recent advances in discovering the biological structure and opening mechanisms of KCa channels, as well as their applications in modulator development. Finally, we elucidate the challenges associated with applying these modulators in clinical practice and suggest potential avenues for the further development of KCa channel-targeting therapeutics. 

## 2. BK Channels

### 2.1. Structure of BK Channels

A BK channel is formed from a tetramer of α subunits that determines the structure of the pore, either alone or with auxiliary BK-β (β1–β4) or BK-γ subunits (γ1–γ4) [20]. The presence of auxiliary subunits varies depending on the tissue type [20]. 

#### 2.1.1. α Subunits of BK Channels

**Figure 1:**
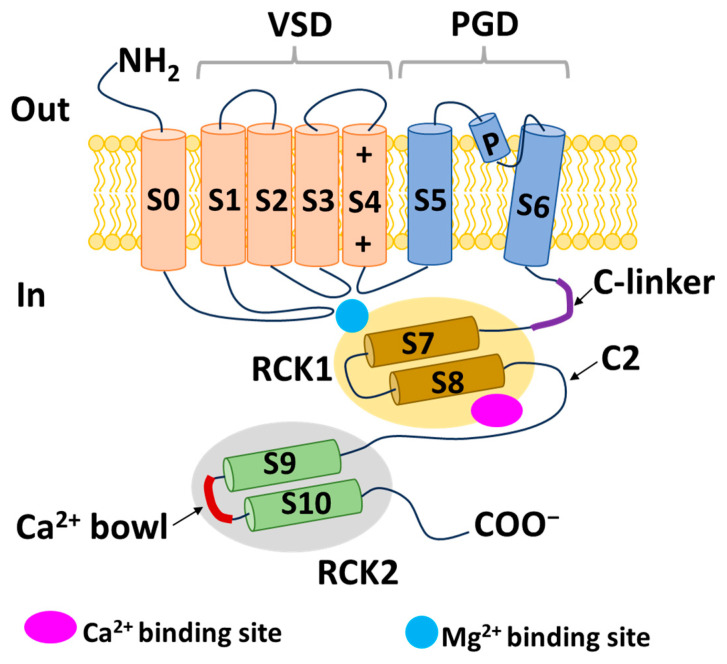
Schematic structure of a BK channel α subunit. VSD, voltage-sensing domain; PGD, pore-gate domain; RCK, regulator of conductance for K^+^; NH_2_, amino terminus; COO^−^, carboxyl terminus; S, segment.

BK channel α subunits include six transmembrane segments (S1–S6), an additional hydrophobic transmembrane segment (S0) near the short external N-terminus, and four intracellular hydrophobic segments (S7–S10) leading to the long C-terminus. Similar to voltage-gated potassium (Kv) channels, the voltage-sensing domain (VSD) of BK channels is structured through the assembly of S1–S4. S4, which harbors positively charged residues (especially Arg213), functions as a voltage sensor [21]. The potassium-selective pore-gate domain positioned at the center is formed by four pairs of S5 and S6 segments and encapsulated by the VSD. In contrast to Kv channels, BK channels contain an extra S0 segment that plays an important role in the interaction between the α and β subunits. In addition, this segment may interact with cross-membrane segments to change the channel’s voltage sensor [22]. In the intracellular C-terminus, the regulators of conductance for K^+^ 1 and 2 (RCK1 and RCK2, respectively) are structured from the S7–S10 segments. The RCK1 region contains Ca^2+^- and Mg^2+^-binding sites, whereas the RCK2 region contains a Ca^2+^ bowl, which includes a string of aspartate residues and plays a role similar to that of the Ca^2+^-binding site of the RCK1 domain [23]. Four RCK1 and four RCK2 domains form two parallel layers to create a “gating ring”, which acts as a Ca^2+^ sensor. The gating ring regulates the opening of BK channels by changing its structure in response to Ca^2+^ [24,25]. The region from the C-terminus of S6 to the N-terminus of RCK1 is known as the C-linker and acts as a pore gate. The BK α subunit structure is described in Figure 1. 

**Figure 2:**
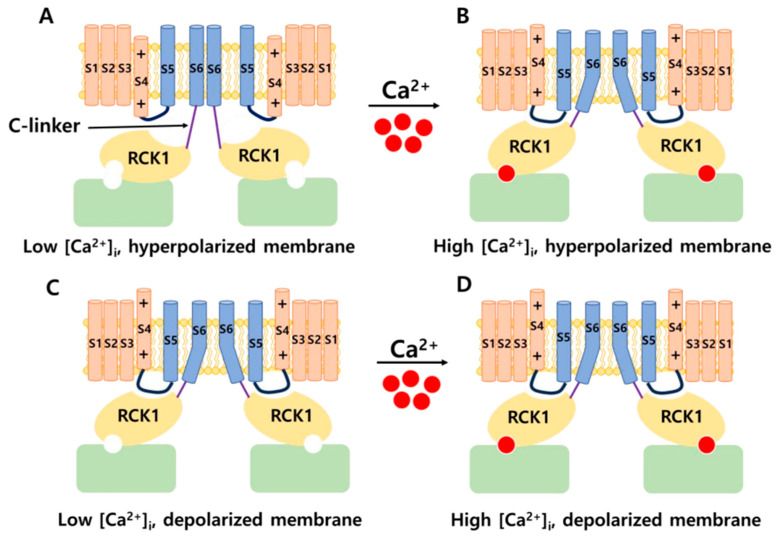
Opening mechanism of BK channels. (**A**) BK channels in their closed state. (**B**) Open state of BK channels in the presence of Ca^2+^. (**C**) Open state of BK channels in a depolarized membrane in the absence of Ca^2+^. (**D**) Open state of BK channels in a depolarized membrane in the presence of Ca^2+^. RCK, regulator of conductance for K^+^; S, segment.

The positions of the voltage sensors and Ca^2+^-binding site in the α subunit have been determined; however, the mechanism by which these sensors activate the channel gate remains unclear. Prior research demonstrated that voltage and Ca^2+^ sensors can function independently to activate BK channels [11,26]. However, substantial evidence indicates that an interaction between the two sensor components influences channel activation [27]. A previous study showed that, in the presence or absence of intracellular Ca^2+^, decreasing or increasing the length of C-linkers enhanced or reduced channel activity, respectively [28]. In addition, a mechanical model was proposed in which the gating ring creates a passive force on S6 through a C-linker without internal Ca^2+^ to regulate the voltage-dependent gating, and the increasing intracellular Ca^2+^ levels modulate this force and open the channel [28]. These findings suggest the existence of interactions between the VSD, C-linker, and gating ring region that impact the channel’s gating ability. In 2017, the cryo-EM structure of a full-length BK channel from *Aplysia californica* was elucidated. Based on the structure of BK channels in the presence and absence of Ca^2+^, the authors proposed an opening mechanism [29,30]. Figure 2 depicts a model of BK channel opening through membrane potential and intracellular Ca^2+^. In the absence of intracellular Ca^2+^, BK channels are closed when the cell membrane is hyperpolarized. In the case of an increase in Ca^2+^ concentration and a hyperpolarized membrane, Ca^2+^ binds to the Ca^2+^-binding site in the RCK1 domain. This has two main consequences. First, it shortens the length of the C-linker, causing a structural change in the S6 segment and pulling it outward from the central pore axis, thereby opening the channel. Second, it causes the voltage sensors to move upward, creating conditions conducive to pore opening. When the membrane is depolarized and the intracellular Ca^2+^ concentration is low, the voltage sensors move upward, causing S4 and S5 to interact with RCK1. This interaction generates a force acting on S6 via the C-linker, opening the BK channel. Simultaneously, changes in the RCK1 region create favorable conditions for Ca^2+^ binding, thereby increasing the affinity of the sensors for Ca^2+^. In the remaining cases, the BK channel opens through both aforementioned mechanisms. This hypothesis explains how membrane voltage influences Ca^2+^ affinity and how Ca^2+^ binding can induce the depolarization of voltage sensors [30].

In addition to the intracellular Ca^2+^ concentration and membrane depolarization, BK channels are further modulated by cytosolic Mg^2+^ and protons. Studies have indicated that Mg^2+^ is involved in the interaction between the VSD and the cytosolic domain to open BK channels [31,32]. Mg^2+^ binds to both the RCK1 domain (Glu374 and Glu399) and the VSD (Asp99 and Asn172) and electrostatically interacts with transmembrane segment S4 (Arg213), leading to enhanced VSD activation [31,32]. In addition, experimentally, Mg^2+^ (10 mM) significantly increased the probability of BK channels opening when voltage sensors were in an activated state but not when they were inactivated [33,34]. When increasing holding potentials from −100 mV to 100 mV, Mg^2+^ increased opening rates in closed BK channels and decreased closing rates in open BK channels [33]. Furthermore, protons may also alter BK channel activity. The region responsible for proton sensing is located within the RCK1 domain (His365 and His394) [35,36]. Reports on the effects of H^+^ on BK channel activity are contradictory. One study showed that an increased intracellular proton concentration inhibited BK currents in smooth muscle cells isolated from small arteries in rat tails [37]. However, another study indicated that protons stimulated BK channels in the absence of Ca^2+^ and Mg^2+^ [38]. The manner in which H^+^ increases BK channel activation has been suggested to resemble the action of Ca^2+^ on the RCK1 domain [35]. Further research is needed to clarify the effect of intracellular protons on BK channel activity. Additional regulatory regions within the C-terminal segment include phosphorylation sites for c-AMP-dependent protein kinase (PKA) and protein kinase C (PKC). The activation and inhibition of BK channels result from phosphorylation by PKA and PKC, respectively [39]. Therefore, metabolic elements contribute considerably to the regulation of BK channel function.

#### 2.1.2. Auxiliary Subunits: BK-β and BK-γ

**Figure 3:**
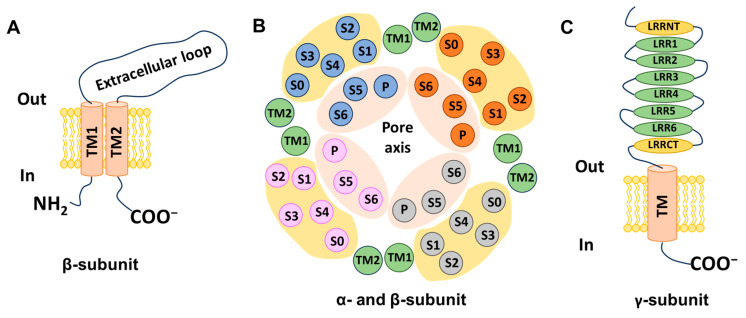
Schematic structure of β and γ subunits of BK channels. (**A**) Structure of β subunits. (**B**) Arrangement of four α subunits and four β subunits viewed perpendicular to the pore axis. The S5 and S6 segments of each α subunit are encapsulated by the VDS of the same α subunit. The TM1 helix is near the S1 and S2 segments, and the TM2 helix is next to the S0 segment. (**C**) Structure of the γ subunits. NH_2_, amino terminus; COO^−^, carboxyl terminus; TM; transmembrane segment; LRR; leucine-rich repeat.

The properties of BK channels are critically shaped by β subunits (β1–β4), which are encoded by *KCNMB1*–*4*, thereby influencing the channels’ physiological roles across various tissue types. Structurally, each β subunit includes two transmembrane helices (TM1 and TM2), an extracellular loop, and intracellular C- and N-terminals, as depicted in Figure 3A [40,41]. The TM2 helix connects to the C-terminal and is adjacent to the S0 segment, whereas the TM1 helix contacts the N-terminal and is close to the S1 and S2 segments of α subunits [42] (Figure 3B). Diversity in the structure and function of BK channels is based on the richness of various auxiliary subunits. When each β or γ subunit combines with an α subunit, it modulates the physiological characteristics of the BK channel to varying degrees. The presence of the BK β1 subunit notably augments the sensitivity to Ca^2+^, reduces voltage sensitivity, and induces the slowing of macroscopic kinetics [43,44]. The β2 subunit also apparently increases Ca^2+^ sensitivity but causes only a non-significant decrease in voltage sensitivity [43,45]. Furthermore, the β2 subunit demonstrates the ability to rapidly and fully inactivate the channel [43,45]. The β3 subunit has four distinct isoforms (β3a–d) formed by alternative splicing, and each of the isoforms exhibits four variants (V1–V4) [46]. The β3a subunit was found to increase sensitivity to voltage, while the opposite effect was found for the β3b subunit [47]. Notably, three subunits (β3a–c) are able to inactivate BK channels incompletely [41,47]. However, in the first study to determine the precise effects of β3d subunits, co-transfection of the β3d subunit was shown not to alter the kinetics of BKα [48]. The neuronal β4 subunit not only substantially retards the kinetics of activation and deactivation but also diminishes perceived calcium sensitivity under low intracellular Ca^2+^ concentrations while conversely heightening the perceived sensitivity under elevated [Ca^2+^]i conditions [49]. It also decreases sensitivity to channel blockers such as charybdotoxin (ChTx) and iberiotoxin (IbTX) [20,49].

Recently, the BK γ1–γ4 auxiliary subunits encoded by LRRC26, LRRC52, LRRC55, and LRRC38, respectively, were identified. The γ subunit features a transmembrane segment accompanied by a substantial extracellular domain constructed from leucine-rich repeat-containing (LRRC) protein, along with a shorter cytoplasmic C-terminal domain. In contrast to β subunits, γ subunits exert a non-significant influence on Ca^2+^ sensitivity and inactivation kinetics. Nonetheless, the four γ subunits possess different modulatory magnitudes to shift the voltage dependence of BK channel activation leftwards by approximately 140 (γ1), 100 (γ2), 50 (γ3), and 20 mV (γ4), even without Ca^2+^ [50,51]. The structure of the γ subunit is illustrated in Figure 3C. 

Auxiliary subunits significantly impact the effects of modulators. Modulators interact with and affect BK channels through the α subunit. However, β subunits can either dramatically enhance or diminish the modulator’s effectiveness on BK channels. Each type of β subunit has different effects and intensities on various regulators. In a previous study, the affinity of ChTX for BK channels containing α and β1 subunits was more than 50 times that for BK channels including only α subunits [52]. Conversely, the toxin association rates of IbTX and ChTX to BK channels consisting of α and β4 subunits were 250–1000 times less than that of channels with α subunits alone [53,54]. Similarly, the β2 subunits also reduce the binding of ChTX to BK channels [40]. Understanding the influences of subunits is therefore crucial for selecting regulators and their concentrations for specific applications.

### 2.2. Distribution and Physiology of BK Channels

Depending on BK channel expression in different subcellular locations, isoforms may manifest distinct physiological and trafficking characteristics determined by auxiliary subunits and alternative splicing [27]. The BK β1 subunit is expressed in vascular smooth muscle in the bladder, kidney, and cerebral artery myocytes but is undetected in the brain [55,56,57,58,59]. The β2 subunit is observed most abundantly in the pancreas, brain, ovaries, kidneys, and spleen [40,57]. The β3 subunit isoforms are located in various organs such as the brain, spleen, pancreas, placenta, heart, kidneys, and lungs [47,59,60]. The brain-specific β4 subunit is predominantly expressed in neuronal tissue [59,61]. It has also been found in smooth muscles of the kidneys and bladder [59,60].

Because of their functional characteristics and widespread expression, BK channels play a pivotal role in the regulation of a diverse array of physiological processes. In the central nervous system, BK channels play a role in regulating the membrane potential of excitable cells, thereby affecting the timing, frequency, and propagation of action potentials (APs) and influencing the release of neurotransmitters [57]. In the heart, BK channels contribute to the repolarization phase of cardiac APs. Alterations in BK channel activity can affect the duration and regularity of heartbeats [62]. BK channels are normally located in the plasma membrane of most cells, except in mature cardiomyocytes, where they localize to the mitochondria [63]. BK channels participate in safeguarding the heart against damage caused by ischemia/reperfusion and enhance the cardioprotective effects of ischemic preconditioning [64]. In the smooth muscle cells of blood vessels, BK channel activation hyperpolarizes the membrane potential, leading to a reduction in Ca^2+^ influx and the relaxation of blood vessels [65]. This mechanism is critical for the regulation of blood pressure and cerebral blood flow [66,67]. BK channels influence the excitability and contractility of the urinary bladder smooth muscle (UBSM) by sustaining the resting membrane potential and molding the repolarization phase of spontaneous APs, which dictate the spontaneous rhythmic contractility of the UBSM [68]. In the respiratory system, BK channels assist in controlling the tone of airway smooth muscles. The activation of BK channels can lead to bronchodilation, facilitating airflow in the lungs [69]. Furthermore, BK channels are associated with various potential physiological functions, including cell metastasis and the activation/migration of non-excitable cells such as fibroblasts [70]. 

### 2.3. Modulators Targeting BK Channels

#### 2.3.1. Neurological Diseases

Epilepsy is a neurological disorder characterized by recurrent and unprovoked seizures. An epileptic seizure is triggered by abnormal synchronous and sustained firing of a group of neurons [71]. Due to the constraining effect of BK channel activation on the depolarization-induced bursting activity within neurons, it has been posited that a reduction in the functionality of BK channels may foster neuronal hyperexcitability, potentially culminating in seizures [72]. Paradoxically, recent findings suggest that certain gain-of-function mutations in BK channels are notably linked to human idiopathic generalized epilepsy, with a particular emphasis on the absence of epilepsy [73,74]. Epilepsy is a threshold phenomenon. Consequently, the optimal pharmaceutical intervention for epilepsy involves a drug that induces only marginal alterations in the seizure threshold [75]. These findings suggest the potential of BK channel modulators for clinical applications in epilepsy. Several BK channel agonists and antagonists have been explored as novel drugs for epilepsy. Zonisamide, a BK channel activator, has been used in combination with other medicines for the management of partial-onset seizures (convulsions) in the clinical treatment of epilepsy since 2000 [76,77]. By modifying the fast inactivation threshold of voltage-dependent sodium (Nav) channels, zonisamide diminishes prolonged, high-frequency, repetitive AP firing [78]. Additionally, zonisamide exerts an inhibitory effect on low-threshold T-type calcium channels within neurons, potentially impeding the propagation of seizure discharges among cellular networks [78]. However, the precise mechanism by which zonisamide exerts its anticonvulsant effects via BK channels remains unclear. Compelling evidence suggests that resveratrol, a phytoalexin naturally occurring in grapes and red wine, serves as an anticonvulsant agent and holds promise as a highly efficacious approach for mitigating neural tissue damage, potentially even preventing the onset of seizures when employed as a complementary component in antiepileptic therapy [79]. Moreover, resveratrol has been shown to enhance the opening activity and the current amplitude of BK channels while concurrently diminishing the amplitude of Nav currents [80]. Given the fundamental roles of BK and Nav channels in the initiation of seizures, these findings imply that the modulation of these channels by resveratrol in cortical neurons likely constitutes a substantial contribution to its antiseizure properties. Paxilline (PAX), a tremorgenic fungal alkaloid and BK channel blocker, has exhibited noteworthy anticonvulsant efficacy, reducing seizure duration and intensity in both picrotoxin and pentylenetetrazole seizure models [81,82]. In addition, IbTX, a specific antagonist targeting BK channels purified from the Eastern Indian red scorpion *Hottentotta tamulus*, inhibits burst activity in primary cultured neurons derived from the cerebral cortex of mice [83]. A previous study indicated that PAX and IbTX successfully reversed pilocarpine-induced alterations in the electrophysiological characteristics of granule cells in an epileptic group [84]. However, their potential clinical utility in the treatment of epilepsy has been hindered by concerns related to toxicity, which has precluded their inclusion in clinical trials. 

Several studies have reported the potential association between a reduction of BK channel activities and Alzheimer’s disease (AD) [85,86]. Amyloid β (Aβ) aggregates were noted to impair the BK channel activities in both the plasma membrane and mitochondria in rodent neurons [87]. Interestingly, the presence of Aβ aggregates is a characteristic of AD [88]. In addition, sulfatides, which are sulfated glycosphingolipids expressed in the central and peripheral nervous systems, could activate BK channels [89]. A decrease in sulfatide levels has been shown to be related to AD [90]. These findings suggested that BK channel activators might be potential candidates for AD treatment. Isopimaric acid, a toxin derived from conifers that opens the BK channel, recovered cognition in an AD mouse model by improving non-spatial memory and synaptic transmission [86].

BMS-204352, a BK channel activator, exhibits positive effects on some neurological disorders. Fragile X syndrome (FXS) is a genetic form of intellectual disability and autism characterized by the inhibition of transcription of FMR1, the gene responsible for encoding the fragile X mental retardation (FMR) protein [91]. BMS-204352 has been shown to restore the glutamate balance within the hippocampus, rectify impairments in social recognition and social interaction, and improve spatial memory [91]. In 2018, Carreno-Munoz et al. showed that BMS-204352 reverses sensory hypersensitivity and prevents the emergence of behavioral abnormalities in Fmr1-knockout mice [92]. These results provide additional support for the theory that BK channels are a molecular target of therapeutic drugs for FXS. Another application of BMS-204352 in improving neurological function is the restoration of impaired habits due to medical conditions. Research suggests that BK channels contribute to habituation via synaptic plasticity [93]. A previous experimental study showed the beneficial effect of BMS-204352 on neuronal ischemia in rats [94]. Interestingly, BMS-204352 was used in a clinical trial called MaxiPost [95]. In phase II of the clinical trial, patients with acute stroke were administered the drug within 48 h of the onset of stroke symptoms. The results indicated no significant difference in adverse effects between the control and treatment groups [95]. However, phase III clinical trials involving 1978 patients at 200 centers worldwide yielded unfavorable outcomes. BMS-204352 did not demonstrate superior efficacy to the placebo in stroke treatment [95]. In 2019, a randomized, double-blind, placebo-controlled, cross-over study found that BMS-204352-activated BK channel activities cause headache and dilate arteries inside and outside the brain in healthy persons. This finding suggested that BK channels might be involved in the pathophysiology of headaches in humans [96].

#### 2.3.2. Cardiovascular Disorders

Several studies have reported that BK channels are promising therapeutic targets in heart rate-related disorders. Recently, the inhibition of BK channels was demonstrated to reduce heart rate both in vitro and in vivo [62,97,98]. In a study by Imlach and colleagues, heart rate was reduced by 70%, 60%, and 42% relative to baseline values using PAX (5 µM), loliterm B (1 µM), and IbTX (0.23 µM), respectively [62]. The mechanism of heart rate reduction due to BK channel inhibition has not yet been elucidated. BK channel inhibitors were reported to reduce heart rate both in vivo and in isolated hearts [62]. This suggests that heart rate reduction might occur due to direct effects on the heart rather than indirect effects on other pathways in the cardiovascular system by BK channel antagonists. Despite their low mRNA expression throughout the heart, BK channels may be expressed to a small degree in certain cell types in the heart [99]. BK channels were reported to be expressed in coronary arterioles; however, potent vasoconstrictors were not shown to significantly affect heart rate in isolated hearts [100]. Therefore, the inhibition of BK channels in coronary arterioles may not be the main mechanism of heart rate reduction. Another study showed that BK channels regulate the firing rate of the sinoatrial (SA) node, which contains specialized cardiomyocytes known as pacemaker cells [97]. BK channel inhibitors prolong the diastolic depolarization phase of the SA cell AP, leading to bradycardia and decreased heart rate [97]. To examine whether plasma membrane BK channels or mitochondrial BK (mitoBK) channels are involved in reducing heart rate, PAX (cell membrane permeable) and IbTX (cell membrane impermeable) were used [62,97,98]. However, the published results were inconsistent. One study showed that both PAX and IbTX reduced heart rate, whereas another study reported that only PAX exerted this effect [97,98]. Therefore, comprehensive and in-depth studies should be conducted to clarify the exact mechanism of the heart rate-lowering effect of BK channel inhibitors.

In contrast with their distribution in other cell types, BK channels are predominantly located within the mitochondrial membrane in adult cardiomyocytes [63]. Several BK channel activators have been developed to improve cardiovascular function and protect against the consequences of cardiac events [64]. In addition to its potential role in preventing seizures by activating BK channels, resveratrol has been studied in the context of cardiovascular protection. Resveratrol improves endothelial function and protects the cardiovascular system by reducing the concentration of endothelin 1 (ET-1), a vasoconstrictor that promotes the hardening of blood vessel cells and stimulates the production of reactive oxygen species (ROS), and increasing the concentration of endothelial NO, a vasodilator that regulates vascular tone, blood pressure, and hemodynamics [101]. Notably, a recent study demonstrated the ability of resveratrol to increase the production of endothelial NO, leading to vasodilation primarily by opening BK channels in the endothelium rather than those in smooth muscle cells [102]. NS1619, a benzimidazole derivative, has been shown to dilate blood vessels through the same intrinsic endothelial BK channels as resveratrol [102]. MitoBK channel activity increases K^+^ conductance and enhances mitochondrial respiratory function by decreasing the generation of ROS and reducing harmful intra-mitochondrial calcium build-up [103]. These processes safeguard the heart against ischemia/reperfusion injury [104]. Several studies have shown that NS1619 protects the heart from ischemia/reperfusion injury in mouse, rabbit, and canine models [105,106,107]. Nonetheless, there is evidence that NS1619 is nonselective. At a high concentration (approximately 100 µM), NS1619 inhibited L-type Ca^2+^ channel activity in rat cardiac muscle cells and Kv channels [108,109]. In addition, NS1619 released Ca^2+^ from internal stores and decreased Ca^2+^ accumulation by the sarcoplasmic reticulum by inhibiting SERCA in H9C2 cells (a cell model similar to cardiomyocytes) [110]. The nonselective effects of NS1619 on cardiomyocytes need to be further investigated to clarify whether it affects the therapeutic potential of this compound. Another agonist that has been studied is NS11021, which demonstrates higher efficacy and specificity than NS1619 [111]. NS11021 increases the channel opening probability by shifting the activation curve of the channel to the left without changing the conductance of the individual channels [111]. Similar to NS1619, NS11021 enhances K^+^ uptake and respiration in the mitochondria, thereby extending the survival of cardiac cells under conditions of local ischemia [103]. However, NS11021 (10 μM) did not appear to exert an influence on several cloned Kv channels and endogenous L-type Na^+^ and T-type Ca^2+^ channels in guinea pig cardiomyocytes [111].

#### 2.3.3. Cancers

BK channels have been reported to participate in cell cycle progression, cell proliferation, and cancer metastasis [112,113,114]. Therefore, BK channel modulators are an attractive potential treatment for cancer. Interestingly, both agonists and antagonists of BK channels have been indicated as anti-cancer agents. 

One study reported that NS1619, a BK channel activator, inhibited the migration of glioma cells independently of intracellular calcium [115]. NS1619 (IC50 = 31.1 µM) was shown to inhibit cell proliferation and induce apoptosis in A2780 cells (an ovarian cancer cell line) [36]. In another study, although *KCNMA1* was overexpressed in triple-negative breast cancer cells when compared to levels in normal breast cells, most BK channels were closed [116]. In a xenograft mice model, treatment with BMS-191011, another BK channel activator, resulted in tumor growth retardation without inducing cardiotoxicity [116].

In neuroblastoma cells, IbTX (0.4 µM) and PAX (50 µM) were reported to induce G1/G2 accumulation and contraction, AKT1pser473 dephosphorylation, and a reduction in cell size. Furthermore, PAX exhibited antiproliferative effects and induced early apoptosis via its action in the nuclear membrane [112]. Past studies showed that BK channels were involved in the migration of glioblastoma cells and that the inhibition of BK channels using PAX (2 µM) and IbTX (100 nM) markedly inhibited cell migration [113,117]. In addition, BK channels were also associated with hypoxia-induced migration and cisplatin resistance in human glioblastoma cells [118,119]. BK channel activities, but not BK channel expression, increased in U87-MG cells under hypoxia, and blocking BK channels using PAX inhibited hypoxia-induced migration and cisplatin resistance [118,119]. BK channels have been reported to be associated with breast cancer cell proliferation, migration, and invasion [120]. Penitrem A, a selective BK channel blocker, was demonstrated to reduce breast cancer cell proliferation and invasion through the Wnt/beta-catenin pathway [114,120,121]. Both IbTX- and tetraethylammonium-mediated inhibition of BK channels led to decreases in cell proliferation, migration, and invasion in hepatocellular carcinoma cells [122]. In ovarian cancer stem cells, trimebutine maleate dramatically suppressed tumor growth in vivo and in vitro through the Wnt/β-catenin, Notch, and Hedgehog pathways by blocking both BK channels and voltage-gated calcium channels [123]. The characteristics and effects of various BK channel modulators are summarized in Table 2.

## 3. IK Channels

### 3.1. Structure of IK and SK Channels

Previously, because of differences in conductivity, it was thought that IK and SK channels did not belong to the same subfamily. After their genetic similarity was elucidated by cloning, IK channels were classified into the KCNN or SK subfamily as KCNN4 or SK4. Because of the significant similarity among the genes encoding the SK and IK channels, the overall topology of these channels is similar. In this section, we outline the structures of IK and SK channels.

**Figure 4:**
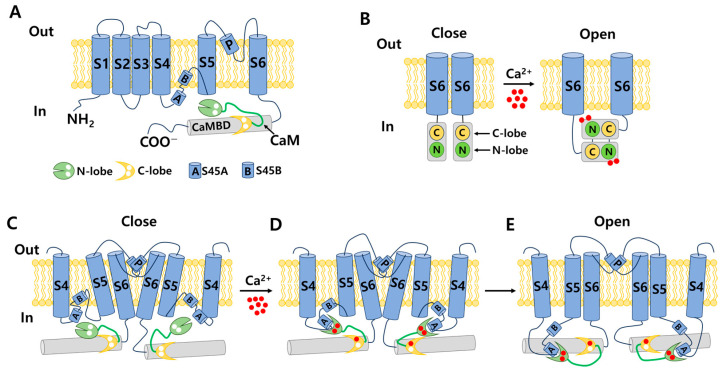
Schematic structure of IK and SK channels and their opening mechanism. (**A**) Structure of IK and SK channels. (**B**) Opening mechanism proposed by Schumacher et al. [127] (**C**–**E**) Opening mechanism proposed by Lee and Mackinnon [19]. CaM, calmodulin; CaMBD, calmodulin-binding domain; NH_2_, amino terminus; COO^−^, carboxyl terminus; S, segment.

Each channel is formed from a four-fold symmetrical tetramer, and each subunit features a common architecture comprising six transmembrane segments (S1–S6) [6,17,125]. The ion channel pore is composed of transmembrane helices S5 and S6 and encircled by membrane-embedded helices S1–S4 originating from the same subunit [19]. This configuration is similar to that of BK channels but diverges from the arrangement found in domain-swapped Kv1–Kv7 channels, wherein helices S1–S4 engage with a neighboring pore domain [19,29]. The S4–S5 linker in the SK and IK channels is distinct from that in the BK channel. In SK and IK channels, it comprises two α-helices (S45A and S45B) as opposed to the shorter turn configuration observed in BK channels [19,29]. Activation of these channels occurs in response to low intracellular Ca^2+^ concentrations (0.1–0.7 μM), facilitated by a mechanism involving the presence of a calmodulin-binding domain (CaMBD) within the channel’s protein structure [15,126]. Each calmodulin molecule is linked to a single subunit, and each calmodulin lobe has a specific role: the C-lobe binds to the CaMBD in a one-to-one ratio independently of Ca^2+^, whereas the N-lobe associates with the S4–S5 linker in response to Ca^2+^ levels [19]. The structures of each subunit are shown in Figure 4A.

In 2001, a CaMBD/Ca^2+^/calmodulin complex with a crystal structure of the SK2 channel and a resolution of 1.60 angstroms was published. The findings demonstrated that the CaMBD/calmodulin complex of each subunit exists in a monomeric state in the absence of Ca^2+^ [127]. In the activation state, the binding of Ca^2+^ to the N-lobes compels the CaMBD/calmodulin monomers to form a structural configuration called the “dimer-of-dimers” structure. This rearrangement pulls the bundle-crossed helices of the pore, opening the channel, as shown in Figure 4B [127]. However, the two-fold symmetry proposed by this dimer-of-dimers configuration is difficult to match to the four-fold symmetry observed in channel pore structures [128]. In 2018, full-length cryo-EM structures of a human SK4–calmodulin channel complex in both the activated and closed states were revealed [19]. Based on this evidence, a model of SK and IK channel activation was proposed, as shown in Figure 4C–E. In the absence of Ca^2+^, the channel is closed, the C-lobe of calmodulin binds to the channel, and the N-lobe interacts weakly with the channel, exhibiting structural flexibility. When the Ca^2+^ concentration reaches the activation threshold, Ca^2+^ binds to the N-lobe, inducing a conformational change that enhances the interaction between the N-lobe and the S45A helix in the S4–S5 linker. Consequently, S45A is displaced away from the channel, leading to a shift in the S45B helix away from the pore axis. This alteration results in a structural change in S6, causing S6 to tilt outward from the channel pore axis, thereby permitting the opening of the channel pore [19]. This hypothesis addresses the long-standing question regarding the gating symmetry of KCa channels.

### 3.2. Distribution and Physiology of IK Channels

IK channels are primarily expressed in blood cells, various immune cells, and secretory epithelial cells. In red blood cells, these channels fulfill a function related to volume regulation, whereas, in lymphocytes, they contribute to the generation of hyperpolarization, a crucial requirement for mitosis and subsequent lymphocyte proliferation [18,129]. In addition to their presence in red blood cells, IK channels are widely observed in various types of leukocytes, including T cells, B cells, mast cells, macrophages, and microglia [18,130,131,132]. IK channels promote T-cell activation and proliferation. Higher expression of IK channels has been observed in activated T cells than in resting T cells [18,133]. The primary function of IK channels in immune cells is to hyperpolarize the cellular membrane and establish the driving force required for calcium entry, which is essential for processes such as cell activation, cell proliferation, and cytokine production [134]. IK channels play a pivotal role in B-cell proliferation and migration. Notably, channel activity increases during the differentiation of activated naïve B cells into memory B cells [130]. Research suggests that the function of IK channels in macrophages is intricately linked with the NF-κB and STAT signaling pathways. In one study, inhibition of these IK channels using TRAM-34 led to the downregulation of NF-κB and STAT3 signaling and hindered the transition of macrophages to their proinflammatory M1 phenotype. Moreover, it reduced the levels of inflammatory factors, including interleukin-1 (IL-1), IL-6, TNF-α, and monocyte chemoattractant protein-1 (MCP-1) [135]. In mast cells, IK channel activation serves to uphold elevated levels of intracellular free Ca^2+^. This activation further facilitates IgE-dependent histamine release and governs the secretory response of mast cells [131]. In addition, IK channels are distributed in secretory epithelial cells of the lungs, colon, pancreas, and salivary glands [136,137,138]. Within the secretory epithelia of the lungs and digestive system, IK channels collaborate with the Na–K–2Cl co-transporter to enable the secretion of chloride (Cl^−^) and fluids [136,137]. In the central nervous system, IK channels are mainly located in the microglia (immune cells) and endothelial cells. Microglial IK channels control several functions, such as respiratory burst, proliferation, migration, and nitric oxide production via lipopolysaccharides [139,140]. In pyramidal neurons, the role of IK channels in the slow after-hyperpolarization (sAHP) phase remains controversial. Some researchers, including Brian King, have demonstrated that Ca^2+^-dependent sAHP is mediated by IK channels [141,142]. It was shown that IK channel agonists (DC-EBIO and SKA-31) increased sAHP, and antagonists (TRAM-34 and senicapoc) reduced sAHP in CA1 pyramidal cells [141,142]. In addition, the synaptically evoked sAHP was decreased in IK knockout mice [141]. However, Kang Wang and colleagues found that TRAM-34 had no significant effect on sAHP or the excitability of CA1 pyramidal neurons [143]. Furthermore, they observed no change in the feature of sAHP current in IK knockout mice [143]. This evidence suggests that IK channels do not mediate sAHP in pyramidal neurons. A review article comparing King and Wang’s studies failed to elucidate the reasons for the dramatic difference in their results [144]. Clarifying the role of IK channels in sAHP is crucial for promoting the development of pharmacological tools for the treatment of diseases related to sAHP. Furthermore, IK channels were reported to be expressed in C2C12 myoblasts and related to myogenic differentiation [145,146,147]. DCEBIO, an agonist of IK and SK channels, enhanced myogenic differentiation in C2C12 cells; this effect was inhibited by TRAM-34 (an inhibitor of IK channels) but not by apamin (an inhibitor of SK channels) [147]. Therefore, IK channels may regulate the muscle differentiation process.

### 3.3. Modulators Targeting IK Channels

#### 3.3.1. Blood Cell Disorders

Owing to the crucial role of IK channels in the physiological activities of red and white blood cells, the regulation of IK channels is a potential target for the treatment of pathological conditions. Senicapoc (ICA-17043) was developed from the structural framework of clotrimazole (an antifungal drug) and entered clinical trials with the goal of treating sickle cell anemia [148]. Senicapoc selectively blocks IK channels, reduces red blood cell dehydration and hemolysis, and increases hemoglobin concentration in sickle cell disease. A phase III double-blind, randomized, placebo-controlled study was conducted to determine the safety and clinical efficacy of senicapoc in 145 patients who received the drug and 144 patients who received a placebo for 52 weeks. The primary endpoint was the difference in the rate of acute sickle cell-related pain crises between the treatment and placebo groups. The acute pains associated with sickle cell disease were specifically defined and were independently and blindly assessed by a committee of five physicians who were experts in sickle cell disease. The acute pain rates were calculated by dividing the total number of qualifying pains recorded by the total number of months in which patients received senicapoc or the placebo. Secondary endpoints were differences in the following factors between groups: (1) number of months from the beginning of treatment until the first, second, and third onset of pain; (2) hematological parameters such as indirect bilirubin, lactate dehydrogenase, reticulocyte and dense erythrocyte counts, hemoglobin, hematocrit, and red blood cell counts. Patients in the treatment group exhibited significantly higher hematocrit and hemoglobin levels and lower density and reticulocyte counts than those in the placebo group. However, the unblinded data monitoring committee terminated this study prematurely and concluded that no discernible effects were observed. Despite achieving the desired biological outcomes, treatment did not reduce clinical pain, a primary efficacy endpoint. No significant difference in the incidence of sickle cell attacks was observed between the two groups. Serious side effects were similar between the two groups. In addition, nausea and urinary tract infections occurred more frequently in the senicapoc group than in the placebo group [149]. Although the results from phase III clinical trials in sickle cell anemia were not sufficient to bring senicapoc to the pharmaceutical market, phase I, II, and III clinical studies have demonstrated that senicapoc is a safe and well-tolerated drug candidate in humans and exhibits biological activity at the doses administered. Therefore, senicapoc has been studied as a potential treatment for other medical conditions. Based on the results of previous studies, senicapoc has been used in clinical trials to treat allergic asthma. In phase II of a clinical trial, senicapoc treatment attenuated elevated airway resistance and decreased exhaled NO, a marker of inflammation [150]. However, in the second proof-of-concept phase II trial examining the effects of senicapoc on patients with exercise-induced asthma, no significant improvement in lung function was observed after four weeks of treatment [150]. Recently, senicapoc was used as a target in drug repurposing for the treatment of hemolytic anemia and xerocytosis, a rare hereditary condition caused by a gain-of-function IK channel mutant [151]. In 2021, an explanatory proof-of-concept study on senicapoc was performed in patients with familial dehydration stomatocytosis induced by a V282M mutation in the IK channel. The study is expected to conclude in March 2024 [152].

Various studies have shown that some pore-blocking inhibitors of IK channels improved pathological conditions in immune-related diseases such as asthma, allergic rhinitis, inflammatory bowel disease, and rheumatoid arthritis [153,154,155,156]. TRAM-34, a well-known IK inhibitor, reduced inflammation in ovalbumin-induced asthma and allergic rhinitis [153,154]. TRAM-34 was designed based on the structure of clotrimazole, and an imidazole group was replaced with a pyrazole group to avoid affecting cytochrome P450 (CYP) activity [157]. However, TRAM-34 was shown to have no inhibitory effect on the human CYP3A4 isoform and was reported to inhibit other CYP isoforms such as human CYP2B6 and CYP2C19 [157]. Currently, TRAM-34 is not used in clinical trials. Nevertheless, because of its highly selective inhibitory properties against IK channels, TRAM-34 is still commonly used in vitro and in vivo to determine the critical role of IK channels in many diseases. In a mouse model of ovalbumin-induced allergic rhinitis, injection of TRAM-34 into the nasal cavity reduced sneezing, nose rubbing, epithelial cell proliferation, eosinophil infiltration, and the expression of IK channels in the nasal mucosa [154]. In addition, in synovial fibroblasts from patients with rheumatoid arthritis, blockage of the IK channel by TRAM-34 diminished cell proliferation and the secretion of proinflammatory cytokines such as IL-6, IL-8, and MCP-1 [156]. Another IK channel inhibitor, NS6180, has been studied in animal models of inflammatory bowel disease. NS6180 inhibited IK channel expression in human, rat, and mouse red blood cells and decreased the levels of cytokines such as IL-2, IL-4, TNF-α, and IFN-γ. NS6180 was demonstrated to be as effective as sulfasalazine, a standard treatment for inflammatory bowel disease, in reducing colitis and improving weight gain [155].

#### 3.3.2. Cancer

TRAM-34 has been used to study the association between cancer and IK channels. Many studies have indicated that IK channels promote cancer progression by influencing cancer cell proliferation, cell cycle progression, invasion, metastasis, and resistance [158,159,160,161]. A previous study indicated that TRAM-34 significantly decreased the proliferation, migration, and invasion of human endometrial carcinoma cells [158]. Another study showed that IK inhibition using TRAM-34 suppressed cell proliferation, migration, and epithelial–mesenchymal transition in triple-negative breast cancer cells [159]. Additionally, TRAM-34 inhibited epithelial–mesenchymal transition and metastasis by increasing E-cadherin expression and decreasing Snail expression in colorectal cancer [162]. Furthermore, the activation of IK channels leads to the hyperpolarization of cell membrane potential, which promotes the G1/S transition in the cell cycle [163]. Hence, the inhibition of IK channels by TRAM-34 arrests the cell cycle at the G0/G1 phase, thereby preventing the growth of endometrial tumors [160]. In addition, TRAM-34 inhibits colorectal cancer progression by inhibiting the secretion of cytokines such as IL-6 and IL-8 by tumor-associated macrophages [164]. TRAM-34 was shown to inhibit migration and invasion induced by CXCL12 and fetal calf serum in glioblastoma cells by inhibiting IK channels [165,166]. Furthermore, TRAM-34 decreased the motility of glioblastoma-derived cancer stem cells, indicating that IK channels are expressed in cancer stem cells deriving from glioblastoma, even though they are not present in tissues from normal neuro and glial cells [167]. Notably, a study reported that TRAM-34 reduced radiation-induced invasiveness in glioblastoma through IK channel inhibition [168]. IK channels may be involved in the metastasis and invasion of glioblastoma cells by modulating cell volume [169]. Hypotonia-induced cell swelling promoted Ca^2+^ influx through mechanosensitive channels, leading to IK channel opening and the recovery of initial cell volume [169]. Other studies indicated that the combination of the IK channel and Orai/STIM channel activities generates Ca^2+^ oscillations, which might induce glioblastoma mobility [170,171,172]. However, the effects of TRAM-34 on cancer cells have been inconsistent. In breast cancer cell line MCF-7, a low concentration (3–10 µM) of TRAM-34 increased cell proliferation, but a higher concentration (20–100 µM) yielded the opposite result [173]. In addition to inhibiting IK channels, TRAM-34 directly interacted with estrogen receptors in a manner similar to 17β-estradiol, thereby enhancing progesterone receptor mRNA expression, reducing estrogen receptor α mRNA expression, and inhibiting the binding of estrogen to its receptor, leading to increased breast cancer cell proliferation [173]. Based on this finding, the use of TRAM-34 as well as other IK channel inhibitors in the treatment of breast cancer should be evaluated with extreme caution. In addition, TRAM-34 (10 µM) unexpectedly enhanced the migration and invasion of pancreatic cancer cells [174]. 

Furthermore, senicapoc suppressed the growth of intrahepatic cholangiocarcinoma cells in a xenograft model in nude mice [175]. Because of its safety and good tolerability, senicapoc is a potential candidate for cancer treatment. 

Therapeutic resistance poses a significant challenge in cancer treatment. Some evidence suggests that 1-ethyl-2-benzimidazolinone (1-EBIO), a nonselective activator of IK channels, promotes the apoptosis of cisplatin-resistant cancer cells [161]. Pillozzi et al. discovered that IK channels affect cisplatin uptake by drug-resistant cancer cells. Therefore, the activation of the IK channel by SKA-31 enhances cisplatin uptake, subsequently promoting apoptosis and inhibiting the proliferation of colorectal cancer cells. This study also indicated that E4031, an inhibitor of Kv11.1, upregulated the expression of IK channels and acted synergistically with cisplatin, similar to SKA-31. The combination of cisplatin, SKA-31, and E4031 yielded maximal effectiveness [176]. Furthermore, in combination with cisplatin, riluzole, a medicine capable of activating IK channels and inhibiting the Kv11.1 channel, overcame drug resistance in colorectal cancer cells [176]. Riluzole has been approved for the clinical treatment of amyotrophic lateral sclerosis in many countries, making it readily available to patients [177]. This combination of drugs holds significant promise for the treatment of drug-resistant cancers. Further clinical studies are required to determine its efficacy precisely.

#### 3.3.3. Neurological Diseases

TRAM-34 and senicapoc exhibit protective effects on the nervous system by reducing damage and attenuating the impairment of function caused by neurological disorders. Blockade of the IK channel using TRAM-34 decreased the symptoms of autoimmune encephalomyelitis in a mouse model of multiple sclerosis [178]. Another study reported that TRAM-34 reduced the activation of microglia/macrophages, leading to a reduction in neuroinflammation related to ischemia/reperfusion stroke [179]. In addition, the inhibition of IK channels with TRAM-34 reduced astrogliosis and microglia activity and improved memory deficits, suggesting that IK channel inhibition may be a promising therapeutic strategy for the treatment of AD [180]. Jin et al. demonstrated the potential of senicapoc in the treatment of AD. Senicapoc can penetrate the brain even when administered orally, decreasing neuroinflammation, reducing the cerebral amyloid load, and improving hippocampal neuroplasticity [181]. These results, combined with the safety evidence in phases I and II of previous clinical trials, support the use of senicapoc in clinical AD treatment. Additionally, in mice with peripheral nerve injuries, senicapoc significantly reduced tactile allodynia without affecting motor activity [182]. In 2022, a clinical trial involving 55 patients lasting 52 weeks was initiated to study the mechanism of action of senicapoc in mild or prodromal AD. This study is expected to end in June 2025 [183]. Table 3 presents an overview of the properties and impacts of IK channel modulators.

## 4. SK Channels

### 4.1. Distribution and Physiology of SK Channels

SK channels are broadly expressed, primarily in the central and peripheral nervous systems and the cardiovascular system [184]. Among the SK channels in the human brain, SK3 is the most highly expressed, followed by SK2 and SK1 [179]. The distributions of SK1, SK2, and SK3 channels in the central nervous system are different. SK1 channels are primarily distributed in the neocortex. Both SK1 and SK2 channels are highly co-expressed in the CA1–3 layers of the hippocampus, thalamic reticular nucleus, cerebellum, and brain stem. SK3 channels are expressed in the midbrain and hypothalamus [177,178,179]. The functioning of SK channels in neurons located in the midbrain and cerebellum plays a role in coordinating muscle movements and facilitating movement [184]. In pyramidal neurons of the hippocampus and amygdala, SK channels modulate the excitatory postsynaptic potential, and the inhibition of SK channels increases long-term potentiation that enhances learning and memory [185].

In neurons, SK currents are responsible for generating the medium phase of AHP, which constitutes the second phase of AHP following an AP. They play a crucial role in regulating intrinsic neuronal excitability and controlling spike firing rates [186]. In addition, Ca^2+^ influx-induced SK channel activation modulates the frequency of AP discharges, leading to the regulation of dendritic excitability. SK currents are typically activated by Ca^2+^ entering the neurons through voltage-gated calcium channels, which are activated during an AP. However, they can also functionally couple with postsynaptic calcium sources, including N-methyl D-aspartate and nicotinic acetylcholine receptors. Additionally, SK currents can be influenced by calcium released from intracellular ryanodine or IP_3_ receptors [187]. This functional coupling to various calcium sources allows SK channels to fine-tune neuronal excitability and synaptic transmission in response to different signaling pathways and neuronal activity patterns. SK channels also play an important role in regulating synaptic transmission [188]. SK channels have been shown to inhibit postsynaptic potentials in dopaminergic neurons in the ventral tegmental area and substantia nigra [188] and to mediate inhibitory postsynaptic conductance in auditory outer hair cells. This occurs after activation by calcium influx through calcium-permeable nicotinic acetylcholine receptors [189]. Furthermore, recent studies have shown that SK channels play a role in shunting fast excitatory synaptic transmission in the lateral amygdala and hippocampal pyramidal neurons [190]. SK channels are involved in regulating synaptic plasticity, a fundamental process underlying learning and memory. Therefore, SK channels may participate in the regulation of learning and memory. Indeed, the blockade of SK channels using apamin in rats resulted in enhanced learning performance in an object recognition task [191]. Additionally, apamin was found to decrease spatial navigation deficits induced by medial septum and hippocampal lesions in mice in the Morris water maze spatial memory task [185]. 

In a normal heart, the SK1 and SK2 channels are primarily located in the atria, whereas the SK3 channel is found in the atria and ventricles [192]. SK channels play an important role in regulating AP duration through a negative feedback system [193]. When intracellular Ca^2+^ increases due to the opening of L-type Ca^2+^ channels or Ca^2+^ release from the sarcoplasmic reticulum, SK channels are activated [194]. Moreover, the opening of SK channels repolarizes cardiomyocytes, leading to the closure of L-type Ca^2+^ channels and the completion of an AP [193]. A longer AP leads to a longer Ca^2+^ influx, thereby increasing SK channel activation and resulting in a shorter AP duration [193]. Indeed, a prolonged AP duration was observed in the atria of SK2-null mice compared to that in control mice, and a decreased AP duration was observed in mice overexpressing SK2 channels [195]. In addition, differences in the distribution of SK channels in the atria and ventricles represent a potential target for the treatment of atrial-selective arrhythmias. SK channels regulate AP duration in the atria; therefore, they play an important role in the pathophysiology of atrial fibrillation (AF). AF is a condition characterized by an irregular and often very rapid heart rhythm. An increase in SK channel activity shortens AP duration and leads to atrial tachypacing. On the contrary, the downregulation of SK channel activity prolongs AP duration, resulting in reduced AF frequency. Interestingly, evidence for both over- and under-expression of SK channels has been provided in models of AF. In a burst-paced rabbit model, the expression of SK2 mRNA and protein was increased in the left atrium [196]. In a dog model, an increase in SK1 and SK2 protein levels and SK2 mRNA expression, but not SK1 mRNA expression, was reported [197]. Posttranslational modifications or altered membrane trafficking of SK1 channels were suggested as the cause of SK1 overexpression [197]. In a dopachrome tautomerase-induces AF model in mice, the mRNA and protein levels of SK1 and SK3 were high [198]. In contrast, a decrease in SK1–3 channel expression was noted in patients presenting AF and heart failure (HF) [199]. In one study, SK2 mRNA expression in the atrial tissue of patients with AF was lower than that in the healthy group [200]. Inconsistencies in the levels of SK channel mRNA and protein may be due to the stage or duration of AF, the pathogenesis model, and differences in atrial tissue among species [193,201]. SK channels might be initially upregulated and then downregulated due to extensive structural and electrical remodeling in the atrium [193,201]. Therefore, SK channel antagonists might be potential candidates for the treatment of AF onset before SK channel expression is downregulated [193]. SK channels are implicated in HF through their role in ventricular tachyarrhythmias [193]. Under normal conditions, SK channels play a negligible role in ventricular tissue. However, SK channels are dramatically upregulated under HF conditions, as observed in a tachycardia-induced HF model in rabbits and in ventricular myocytes isolated from end-stage HF patients [202,203,204]. In addition, apamin (an inhibitor of SK channels) showed significant prolongation in AP duration in failing ventricles but no significant effect in normal ventricles [202,205]. Hence, SK channel inhibitors seem to be promising for HF treatment. However, adjusting heart rate under HF conditions is very complicated, so the use of SK channel modulators for HF treatment must be considered [205]. SK channels are related to hypertension through negatively modulated intracellular Ca^2+^ concentration and aldosterone secretion [206]. Apamin, a well-known selective SK channel blocker derived from bee venom, inhibits SK channels and increases Ca^2+^ and aldosterone levels, resulting in hypertension. Consistently, DCEBIO, which antagonized the actions of apamin, showed the opposite effects [206].

### 4.2. Modulators Targeting SK Channels

#### 4.2.1. Neurological Diseases

As shown above, both SK channel upregulation and downregulation may be involved in correcting nervous system imbalances. Therefore, SK channel agonists and antagonists have been investigated as potential therapeutic agents for neurological disorders. Several studies have suggested that 1-EBIO, a nonselective activator of SK channels, reduces acoustically evoked seizures in both male and female genetically epilepsy-prone rats [207]. In addition, 1-EBIO suppressed epileptiform activity in an acute hippocampal slice model [208]. 1-EBIO also decreased seizure probability and increased the threshold for pentylenetetrazole-induced seizures in mice [209]. DCEBIO, a derivative of 1-EBIO, modulates fear extinction memory by upregulating the SK potassium channels in the infralimbic cortex [210]. Another SK channel activator, NS309, has been reported to exert a neuroprotective effect through SK channels. In a rat model of traumatic brain injury, NS309 significantly decreased brain edema, alleviated deficits in neurological function, and attenuated neuronal apoptosis [211]. This neuroprotective effect was mediated through anti-inflammatory and immunomodulatory mechanisms [211]. In addition, activation of SK channels by NS309 confers a protective effect on human dopaminergic neurons, ameliorating the dopaminergic cell depletion that predisposes patients to Parkinson’s disease [212]. Apamin has been shown to have a positive effect on learning and memory in vitro. Blocking SK channels using apamin increases the excitability of hippocampal neurons and induces synaptic plasticity, which is thought to underlie memory formation [213]. Additionally, researchers have suggested that apamin may enhance object recognition memory, improve the retrieval of extinction memories, and enhance cognitive function when memory declines [210,214,215]. 

Some medicines licensed for the treatment of neurological diseases have been reported to be associated with SK channel regulation. Chlorzoxazone (CZX) was approved by the Food and Drug Administration as a muscle relaxer to treat pain during muscle spasms [216]. Although the precise mechanism of action of CZX has not been completely clarified, evidence suggests that it triggers SK2 channel activation [217,218]. One study suggested that CZX may positively modulate SK channels, leading to reduced neuronal activity [219]. In addition, CZX was proposed as a new therapy for episodic ataxia type 2, an inherited movement disorder caused by mutations in the gene encoding the CaV2.1 α1 subunit [220]. As mentioned above, riluzole has been approved for the treatment of amyotrophic lateral sclerosis in most nations [177]. The precise biochemical target of riluzole in motor neuron disease remains uncertain; however, the pharmacological targets of riluzole include SK channels [221]. Based on its ability to activate SK channels, riluzole has been shown to be effective in ameliorating disease-related loss-of-function defects in an animal model of spinal muscular atrophy and inhibiting pain behavior in a mouse model of joint pain [221,222]. Positive outcomes associated with riluzole have also been documented in the treatment of spinocerebellar ataxia type 2, a condition characterized by abnormal firing patterns and the eventual death of Purkinje cells [223]. The effect of riluzole appears to be mediated by the activation of the SK2 channel, which is highly expressed in Purkinje cells [224].

#### 4.2.2. Cardiovascular Diseases

Since SK channels, especially SK1 and SK2, are chiefly expressed in the atrium, they are considered potential targets for AF and arrhythmia treatment. The SK channel inhibitor N-(pyridin-2-yl)-4-(pyridin-2-yl)thiazol-2-amine (ICA) showed antiarrhythmic properties by inhibiting SK channels directly and Nav channel indirectly [225]. ICA extends the AP duration and transfers the resting membrane potential to more depolarized potentials, resulting in the slowing of conduction and a reduction in excitability [225]. Furthermore, the combination of ICA and dofetilide (a class III antiarrhythmic agent that blocks potassium current) or amiodarone (an antiarrhythmic medication and potassium blocker) at a sub-efficacious dose showed a protective effect against AF and reduced the risk of ventricular arrhythmias [226,227]. Other combinations of ICA and flecainide (a drug for abnormally high heart rate treatment and a fast sodium current blocker) or ranolazine (a medication for treating heart-related chest pain and a late sodium current inhibitor) at sub-efficacious concentrations have been studied to examine the synergistic effects of AF treatment [228]. Positive outcomes have been documented, with a reduction in AF duration in a pig heart model [228]. AP14145, a negative modulator of SK2 and SK3, extended the atrial effective refractory period without acute triggers in the central nervous system of mice [229]. AP14145 was also suggested to potentially cure vernakalant-resistant AF in a pig model [230]. Additionally, in an experimental porcine model simulating obstructive respiratory events, AP14145 successfully mitigated the abbreviated atrial effective refractory period associated with intermittent negative airway pressure exposure, reduced the susceptibility to AF, and maintained ventricular electrophysiological function [231]. In addition, apamin, NS8593 (a Ca^2+^-desensitizing modulator), and UCL1684 (an SK channel blocker) have been studied in various AF models [232]. While apamin showed no antiarrhythmic effect, the two remaining substances yielded a dramatic prolongation of the atrial effective refractory period and a reduction in AF [232,233]. The role of SK channels in regulating heart rate has been only partially explored. Modulators of SK channels appear to both terminate and induce arrhythmias. Additional research is imperative to formulate medications and evaluate the impact of these pharmaceutical agents on the susceptibility to arrhythmias. Finding a balanced impact range is crucial. The combination of SK channel modulators with recognized antiarrhythmic medication has shown positive results and is a potential direction. Information regarding SK channel modulators is presented in Table 4.

## 5. Discussion

KCa channels are widely distributed in the body and play various physiological and pathological roles. Numerous studies on KCa channel activators and inhibitors have been conducted in vitro, in vivo, and in clinical trials to identify novel pharmaceutical therapies. However, endeavors to develop KCa channel-targeting drugs have not yet translated to the widespread clinical application of KCa channel modulators. In this section, we discuss reasons for the limited progress in the therapeutic development of KCa channel modulators and propose novel strategies to advance this field.

There are several reasons why few KCa channel modulators have been introduced to the pharmaceutical market. First, some modulators have not demonstrated significant efficacy in clinical treatment. As described earlier, BMS-204352, a BK channel agonist, successfully passed through phase II of a clinical trial; however, it did not show superior efficacy when compared with a placebo in the treatment of acute stroke in a phase III clinical trial [95]. Senicapoc (ICA-17043), an IK channel antagonist, significantly improved biological outcomes in patients with sickle cell disease in a phase III clinical trial but did not alleviate clinical pain. Additionally, more frequent side effects such as nausea and infection were observed in the treatment group than in the control group. Consequently, this clinical trial was prematurely terminated [149]. Secondly, due to their potent pharmacological properties, which can pose serious risks to patients, modulators like PAX, IbTX, and apamin have not been used despite their high selectivity. PAX was reported to cause tremors that lasted for several hours in mice with an ED50 of 25 mg/kg of body weight [234]. It was less toxic than other tremor-inducing drugs, with an LD50 of 150 mg/kg in mice [234]. PAX induced shorter and less serious tremors than loliterm B in mice and other vertebrates [235,236,237,238]. IbTX was purified from the Eastern Indian red scorpion *Hottentotta tamulus.* This scorpion’s toxin could simultaneously inhibit K^+^ channels and activate Na^+^ channels, and a prolonged effect causes the release of a large amount of catecholamines, leading to vasoconstriction and increased blood pressure [239,240]. In some severe cases, IbTX induces pulmonary edema, tachycardia, and myocardial failure [239,240]. Apamin is a small peptide (18 amino acids) that can pass through the blood–brain barrier [241,242]. However, some evidence has suggested the neurotoxicity of apamin. Apamin treatment led to unconstrained polysynaptic spinal reflexes in cats [243]. In addition, the injection of apamin induced convulsions and long-lasting spinal spasticity in mice [243,244]. It also caused tremors, ataxia, and dramatic hemorrhage in the lungs in mice [244]. Recently, lethal dose values of apamin in mice have been investigated: the LD50 for intravenous and intracerebral administration are approximately 4 mg/kg and 1.8 µg/kg body weight, respectively [245]. To our knowledge, the toxicity of PAX, IbTX, and apamin in humans remains unknown. This has hindered investigators and physicians from using these substances clinically. Researchers have made efforts to synthesize small-molecule substances that are both highly selective and have low toxicity based on the structures of these modulators. However, the lack of three-dimensional structural information on these modulators in the resting and active states, along with structural changes upon interactions with other molecules, pose significant challenges to the design of novel modulators. Finally, several modulators may exhibit unintended effects. For example, modulators could induce off-target effects, leading to serious adverse events. TRAM-34, an IK channel antagonist, inhibited the activites of some human CYP isoforms, including CYP2B6 and CYP2C19, with IC50 values of 0.9 µM and 1.8 µM, respectively [157]. In addition, TRAM-34 stimulated and inhibited human CYP3A4 activities when using different substrates (7-benzyloxy-4-[trifluoromethyl]coumarin and dibenzyl fluorescein) [157]. The induction or inhibition of CYP enzymes not only affects the metabolism of TRAM-34 itself but also creates potential CYP-related drug–drug interactions [157]. In a clinical trial, BMS-204352 induced headaches and the dilation of intracerebral and extracerebral arteries [96]. In this study, healthy volunteers received an intravenous infusion of 0.05 mg BMS-204352 per minute or a placebo for two different days [96]. BMS-204352 significantly increased the number of people with headaches, their headache intensity, and the diameter of the superficial temporal artery and peripheral artery [96]. Based on this finding, attention must be paid to side effects related to headaches and arterial dilation when using BMS-204352 in clinical practice.

Despite these challenges, new tactics for applying KCa channel modulators in clinical treatments are being researched and published. First, licensed drugs or modulators that previously failed in clinical trials have been repurposed for other indications. Trimebutine maleate is an approved medicine with antimuscarinic and weak mu-opioid agonist effects and is used for the treatment of irritable bowel syndrome and gastroesophageal reflux disease [246]. Recently, trimebutine maleate was studied for the treatment of ovarian cancer and has shown positive results in preventing ovarian cancer recurrence and drug resistance both in vivo and in vitro via BK channel inhibition [123]. Furthermore, following its failure in sickle cell anemia treatment trials, senicapoc is currently undergoing clinical trials for the treatment of dehydrated stomatocytosis and AD [152,183].

Additionally, KCa channel modulators can be combined with approved medicines to potentially lower the required dosages, limit side effects, and overcome drug resistance. For example, Kirchhoff et al. combined ICA with amiodarone or dofetilide to treat AF [226]. Amiodarone is commonly used for AF prevention; however, at therapeutic doses, it can cause serious side effects such as pulmonary fibrosis, thyroid dysfunction, and neurological disorders and may lead to prolonged QT intervals [247]. Dofetilide is a class III antiarrhythmic drug that is considered an alternative to amiodarone in high-risk patients with AF. However, dofetilide is known to prolong the QT interval, which can increase the risk of torsades de pointes arrhythmias, which are potentially fatal [248]. The drug combination yielded relatively promising results, even when concentrations lower than the therapeutic doses of each individual drug were used. The combination of ICA and amiodarone reduced AF duration, and the combination of ICA and dofetilide reduced AF duration without prolonging the QT interval [226]. Another study showed that combining cisplatin with SKA-31 (an IK channel activator) and E4031 (a Kv11.1 channel inhibitor) promoted apoptosis and inhibited proliferation in cisplatin-resistant colorectal cancer cells. The activation of IK channels and inhibition of Kv11.1 channels led to increased IK channel activities and enhanced cisplatin uptake by cells. Indeed, the combination of cisplatin and riluzole, which could activate IK channels and inhibit Kv11.1 channels, also overcame cisplatin resistance in colorectal cancer. Further studies should be conducted to investigate whether riluzole treats cisplatin-resistant conditions in other cancers. Moreover, riluzole is an approved medication; therefore, it has the potential to be applied to clinical treatment [176]. 

Finally, efforts to identify novel KCa channel modulators are ongoing through the screening of existing compounds. Screening has been performed through two major approaches: (1) high-throughput screening and (2) in silico screening. High-throughput screening uses various assays such as ligand binding, 86Rb^+^ flux, voltage-sensitive dye, and Tl^+^ flux assays [249]. Given the advances in the structural discovery of KCa channels, in silico screening also has great potential for novel drug discovery. Recently, an allosteric modulator of BK channels named BC5 was discovered using in silico screening [250]. BC5 was found to meet the following criteria: (1) it interacts with the interface of the intracellular tail and VSD, and (2) it opens BK channels through a Ca^2+^-dependent pathway [250]. Interestingly, BC5 activated the channel in the absence of Ca^2+^, but Ca^2+^ binding inhibited the effect of BC5 [250]. Further modification of the BC5 chemical structure may reveal new compounds with stronger allosteric activation activities on BK channels. 

## 6. Conclusions

KCa channels are potential therapeutic targets due to their diverse and essential roles in various pathological conditions. Therefore, KCa channel modulators have been extensively studied despite the challenges in their clinical implementation. Over the past two decades, remarkable advancements have been made in the development of compounds targeting KCa channels for treatment. However, more efforts are still needed to bring Kca channel modulators into clinical practices. Currently, drug repositioning for new indications is a time- and cost-saving strategy that aligns well with the diverse physiological roles of KCa channels. Coordinating drugs that have common targets and synergistic effects to reduce dosage and toxicity is also an attractive option. Furthermore, the design and development of new modulators are promising due to the newly discovered cryo-EM structures of KCa channels.

## Figures and Tables

**Table 1:** Characteristics of KCa channels.

	BK Channel	IK Channel	SK Channel (SK1, SK2, SK3)
Opening mechanism	Voltage-dependent and Ca^2+^-dependent	Only Ca^2+^-dependent	Only Ca^2+^-dependent
Conductance (pS)	200–300	30–40	4–14
Aliases	KCa1.1, Maxi-K, Slo1	KCa3.1, SK4, IK1	KCa2.1, KCa2.2, KCa2.3
Ca^2+^-binding affinity (μM)	1–11	0.2–0.5	0.3–0.6
Ca^2+^-binding site	RCK domain	Calmodulin-binding domain	Calmodulin-binding domain
Gene encoding α-subunit (human)	*KCNMA1*	*KCNN4*	*KCNN1*, *KCNN2*, *KCNN3*

**Table 2:** Summary of potential modulators of BK channels.

Modulator/Drug	Structure	Characteristics of Modulator/Drug	Effects on Diseases or Disorders	References
Zonisamide	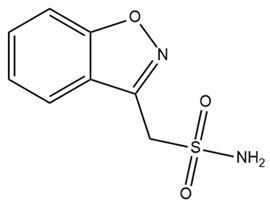	BK activator Nav and T-type Ca^2+^ blocker Approved drug used to treat symptoms of epilepsy and Parkinson’s disease	Combined with other medicines to manage convulsions The main anticonvulsant mechanism via BK channel is unknown	[76,77,124]
Resveratrol	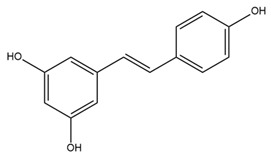	BK activator, Nav inhibitor Phytoalexin naturally occurring in grapes and red wine	Reduces nerve tissue damage Prevents the onset of seizures Decreases ET-1 and increases NO through mitoBK Protects cardiovascular system	[64,79,80,101]
Paxilline (PAX)	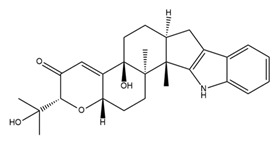	BK channel blocker Tremorgenic fungal alkaloid	Reduces seizure duration and intensity in epilepsy Reduces heart rate Reduces firing rate in SANC and heart rate Induces cell cycle arrest and reduces cell size, migration, invasion, and induction of apoptosis	[62,81,82,97,98,112,113]
Iberiotoxin (IbTX)	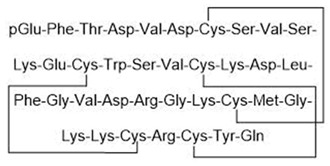	BK channel blocker Purified from scorpions	Inhibitory effects on burst activity in primary cultured mouse neurons Reduces heart rate Induces cell cycle arrest and decreases cell proliferation, migration, and invasion	[62,83,84,97,98,112,113,122]
Isopimaric acid (ISO)	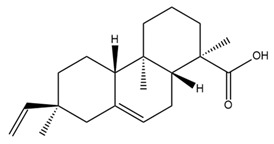	BK activator Toxin derived from conifers	Cognitive recovery in mouse AD model	[86]
BMS-204352 (MaxiPost)	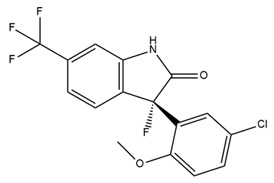	BK and Kv7 activator Phase III clinical trial for acute ischemic stroke treatment	Restored glutamate balance in the hippocampus and improved spatial memory in a rat FXS model Restores habits that are impaired due to illness Improves ischemia in nerves	[91,92,93,94,95]
Lolitrem B	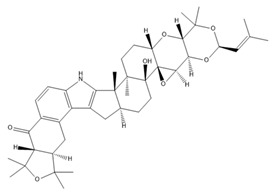	BK inhibitor	Reduces heart rate	[62]
NS1619	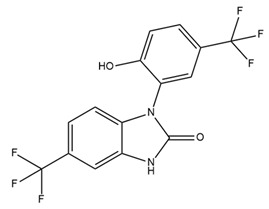	BK activator Benzimidazole derivative L-type Ca^2+^ channel and Kv channel inhibitor	Dilates blood vessels through mitoBK Cardioprotective effects against ischemia/reperfusion injury Reduces cell proliferation, migration, and induction of apoptosis	[36,102,104,115]
NS11021	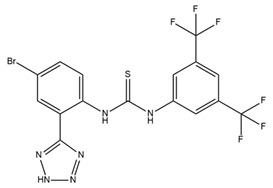	BK activator	Increases channel opening likelihood Extends the survival of cardiac cells under locally ischemic conditions	[103,111]
BMS 191011	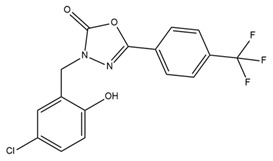	BK activator	Suppresses tumor growth	[116]
Penitrem A	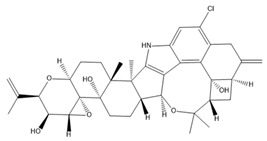	BK inhibitor	Reduces cell proliferation and invasion	[114,120,121]
Tetra-ethylammonium	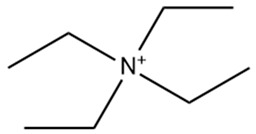	BK inhibitor	Decreases cell proliferation, migration, and invasion	[122]
Trimebutine maleate	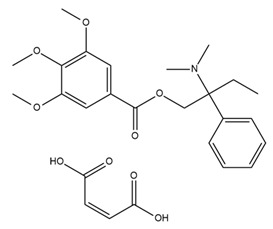	BK and voltage-gated calcium channel inhibitor	Suppresses tumor growth	[123]

**Table 3:** Summary of potential modulators of IK channels.

Modulator/Drug	Structure	Characteristics of Modulator/Drug	Effects on Diseases or Disorders	References
Senicapoc (ICA-107043)	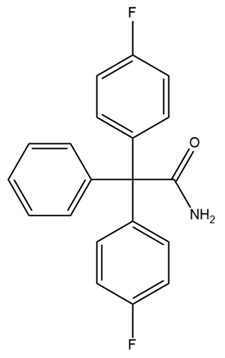	IK-selective inhibitor Developed based on clotrimazole structure Phase III clinical trial in sickle cell anemia patients	Reduces red blood cell dehydration and hemolysis and increases hemoglobin concentration in sickle cell disease without decreasing clinical pain Attenuates the increase in airway resistance and decreases an exhaled inflammation marker (NO) Reduces tactile allodynia without any effect on motor activity Suppresses the growth of intrahepatic cholangiocarcinoma cells	[149,150,175,182]
TRAM-34	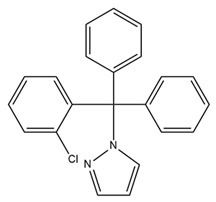	IK-selective inhibitor	Reduces the activation of microglia/macrophages, leading to a reduction in neuroinflammation related to ischemic/reperfusion stroke Improves memory deficits in AD Decreases sneezing, nose rubbing, epithelial cell proliferation, eosinophil infiltration in allergic rhinitis Diminishes cell proliferation and the secretion of proinflammatory cytokines in synovial fibroblasts from rheumatoid arthritis patients Inhibits cancer cell proliferation, cell cycle, invasion, metastasis, and resistance	[154,156,160,161,179,180]
NS6180		IK-selective inhibitor	Decreases IK expression in human, rat, and mouse red blood cells Reduces cytokines (IL-2, IL-4, TNF-α, and IFN-γ)	[155]
1-EBIO	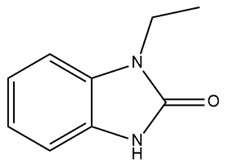	IK and SK activator	Promotes the apoptosis process of cisplatin-resistant cancer cells	[161]
SKA-31	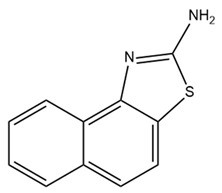	IK activator	Combined with E4031 and cisplatin, promotes apoptosis and inhibits the proliferation of colorectal cancer cells	[176]
Riluzole	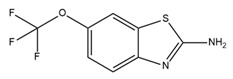	IK and SK activator	Combined with cisplatin, overcomes drug resistance in colorectal cancer cells	[176]

**Table 4:** Summary of potential modulators of SK channels.

Modulator/ Drug	Structure	Characteristics of Modulator/Drug	Effects on Diseases or Disorders	References
1-EBIO	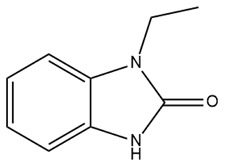	SK and IK activator	Reduces acoustically evoked seizures Suppresses epileptiform activity in the acute hippocampus Decreases seizure probability in mice	[207,208,209]
DCEBIO	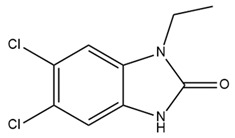	SK and IK activator	Modulates fear extinction memory	[210]
NS309	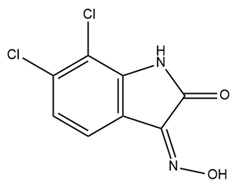	SK and IK activator	Neuron protection role Decreases brain edema, traumatic brain injury-induced deficits in neurological function, and neuronal apoptosis Improves dopaminergic cell depletion	[211,212]
Apamin	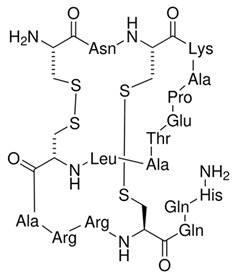	SK-selective inhibitor Derived from bee venom	Increases the excitability of hippocampal neurons and induces synaptic plasticity Enhances object recognition memory and improves the retrieval of extinction memories	[210,213,214,215]
Chlorzoxazone (CZX)	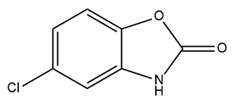	SK2 activator Approved drug to treat muscle pain	Reduces neuronal activity and ultimately results in decreased alcohol consumption Episodic ataxia type 2 treatment	[216,219,220]
Riluzole	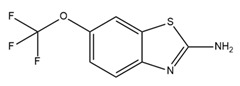	SK and IK activator Approved for amyotrophic lateral sclerosis treatment	Improves disease-related loss-of-function defects in a mouse model of joint pain Used in spinocerebellar ataxia type 2 treatment	[221,222,223]
ICA	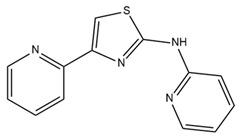	SK inhibitor	Prolongs AP duration and reduces excitability Combined with dofetilide or aminodarone to treat AF, reduces the risk of ventricular arrhythmias with fewer side effects Combined with flecainide or ranolazine, reduces AF at sub-efficacious doses	[225,226,227]
AP14145	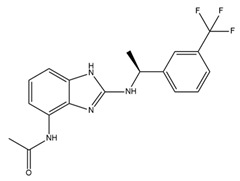	SK2 and SK3 inhibitor	Extends the atrial effective refractory period Attenuates vernakalant-resistant AF in a pig model Improves obstructive respiratory events	[229,230,231]
NS8593	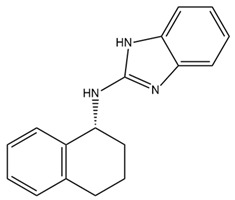	SK inhibitor	Prolongs the atrial effective refractory period and decreases AF	[233]
UCL1684	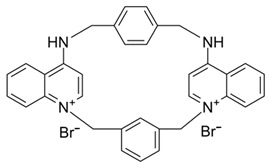	SK inhibitor	Prolongs the atrial effective refractory period and decreases AF	[233]

## Data Availability

The data, analytic methods, and study materials that support the findings of this study are available from the corresponding author upon reasonable request.
